# Microsurgical Reconstruction of the Jaws Using Vascularised Free Flap Technique in Patients with Medication-Related Osteonecrosis: A Systematic Review

**DOI:** 10.1155/2018/9858921

**Published:** 2018-06-07

**Authors:** Roberto Sacco, Nicola Sacco, Umar Hamid, Syed Hasan Ali, Mark Singh, John St. J. Blythe

**Affiliations:** ^1^Barts and The London School of Medicine and Dentistry, London, UK; ^2^Eastman Dental Institute, London, UK; ^3^King's College Hospital, London, UK; ^4^Department of Anaesthesiology, Resuscitation and Intensive Care Medicine, University of Campania “Luigi Vanvitelli”, Naples, Italy; ^5^Mid Essex Hospital Service NHS Trust, Chelmsford, UK; ^6^Bart's and The London NHS Trust, London, UK

## Abstract

**Background:**

Osteonecrosis of the jaw (ONJ) has been reported to be associated with patients receiving primarily bisphosphonate (BP) therapies. However, lately it has been documented that other medications, such as RANK ligand inhibitor (denosumab) and antiangiogenic drug, can cause ONJ. Micro-osseous-vascular reconstruction of the jaws in patients affected by medication-related osteonecrosis of the jaw represents a viable option of treatment for patients affected by stage III of the disease. However, there are still considerable doubts about the success of this procedure in the short, medium, and long term.

**Material and Methods:**

A multidatabase (PubMed/MEDLINE, EMBASE, and CENTRAL) systematic search was performed. Any type of studies considering human patients treated with antiresorptive and antiangiogenic drugs was considered. The aim of the research is to primarily understand the success rate of micro-osseous-vascular reconstruction in the short, medium, and long period of time. This review has also the goal of better understanding any perioperative and postoperative complications resulting from the use of the reconstruction techniques.

**Results:**

Eighteen studies resulted eligible for the study. Fibula free flap is the most commonly utilised vascularised free flap reconstruction technique (80.76%). Ten out of eighteen studies reported no complications. Recurrence of osteonecrosis was registered in five cases (6.41%) after free flap reconstruction. The overall free flap success rate was 96.16%.

**Conclusions:**

Based on the limited data available in literature (Level 4 of the Oxford Evidence-based medicine scale), micro-osseous-vascular reconstruction of the jaws represents a valid treatment in patients with bisphosphonate-related osteonecrosis at stage III of the disease. However, additional data based on a larger cohort of patients are necessary to justify this type of intervention in patient affected by MRONJ.

## 1. Introduction

Bisphosphonates (BP) are antiresorptive drugs used in the management of conditions as diverse as osteoporosis and metastatic bone diseases. These drugs are widely administered and generally well tolerated by patients. In 2003, Marx et al. [1] first reported a nonhealing necrosis of the maxillofacial region in some patients taking BPs.

In the last decade researchers have discovered that BPs not exclusively cause osteonecrosis of jaws, as other drugs, such as antiresorptive (bone-targeted) agents like denosumab, but also were found to cause it. In addition, monoclonal antibodies able to bind and selectively inhibit VEGF-A, specifically mTOR inhibitors, can also cause osteonecrosis of the jaw [2–6].

For this reason, in 2014 the bisphosphonate-related osteonecrosis of the jaw (BRONJ) nomenclature was changed by the position paper of the American Association of Oral and Maxillofacial Surgeons (AAOMS) special committee on Medication-Related Osteonecrosis of the Jaws (MRONJ) [7]. The term “medication-related osteonecrosis of the jaws” (MRONJ) refers to a complication associated with groups of medications, such as antiangiogenic or antiresorptive drugs [8]. These medications can have different indications depending on their mode of administration (Tables 1 and 2) [9, 10].

According to AAOMS, MRONJ is defined as an exposition of necrotic bone in the oral cavity lasting more than 8 weeks, in patients who took antiresorptive or antiangiogenic drugs; these patients have not been exposed to head and neck radiotherapy, nor show signs of bone metastases in the maxillofacial region [7].

A number of systemic risk factors have been associated with increased likelihood of MRONJ; they are summarised in Table 3 [11, 12].

Dental extraction or other surgical procedures such as apicectomies or cystectomies have been found in between 52% and 80% of MRONJ patients' medical history [13–15].

During the last decade AAOMS has revised and proposed a clinical staging classification system of the disease in an attempt to guide clinicians and surgeons to an appropriate therapeutic approach (Table 4).

The management of MRONJ is reported to be very challenging and with no current “gold standard”. Published studies have reported a number of approaches to treatment, with widely varying success rates, ranging from no or limited to radical surgery. The ideal outcome is total eradication of MRONJ along with an improvement of patients' quality of life through pain release and infection management [16].

Conservative treatment was considered to be partially successful, with resolution reported in only 50% of cases; particular concerns have been reported on MRONJ at clinical stages II and III [17–19]. In case conservative treatments fail, surgical approaches like local debridement, osteoplasty, and segmental osteotomy are normally performed [20, 21].

However, patients that show evidence of MRONJ stage III with severe pain, infection, pathologic fracture, extra-oral fistula, or osteolysis extending to the inferior border of the mandible require an invasive type of surgery which might result in a disabling outcome [7, 16, 22].

The absence of a well-established surgical treatment protocol in scientific literature makes it difficult to conduct therapy in advanced cases of the disease.

Up to date, there is no standard treatment for MRONJ associated with antiresorptive and antiangiogenic therapies. Several treatment options have been described since MRONJ was first reported. Although the initial stages of MRONJ seem to respond quite well to conservative treatments or limited bone debridement if conservative treatment fails, the treatment for stage III lesions remains still controversial [23, 24].

The objective of this review is to evaluate the outcome of free vascularised osseous tissue transfer and/or osteofasciocutaneous free flap as treatment for patients affected by MRONJ stage III. Systematic reviews have been already published. However, these reviews were not performed in a standardised manner or did not follow strict criteria. Moreover the previous reviews did not consider antiangiogenic drugs in the search criteria. This has resulted in lack of quality assurance, summarised in Table 5 [25–27]. This review aims to improve the quality of previous research and expand on the current data available.

## 2. Materials and Methods

This systematic review was performed according to PRISMA guidelines [44].

The following the databases were used for the review: PubMed/MEDLINE, EMBASE, and Cochrane Central Register of Controlled Trials (CENTRAL). A three-stage screening approach was used to ensure precision and the quality of the search. The screening of titles and abstracts was carried out independently by three authors (AH, UH, and RS) to eliminate any irrelevant materials (i.e., reviews, animal studies, nonclinical studies, and studies that did not report on patients undergoing to free tissue graft). Disagreements were resolved by discussion.

A data screening and abstraction form was used to

(1) verify the study eligibility derived from the above inclusion/exclusion criteria,

(2) carry out the methodological quality assessment,

(3) extract data on study characteristics and outcomes for the included studies.

**Figure 1:**
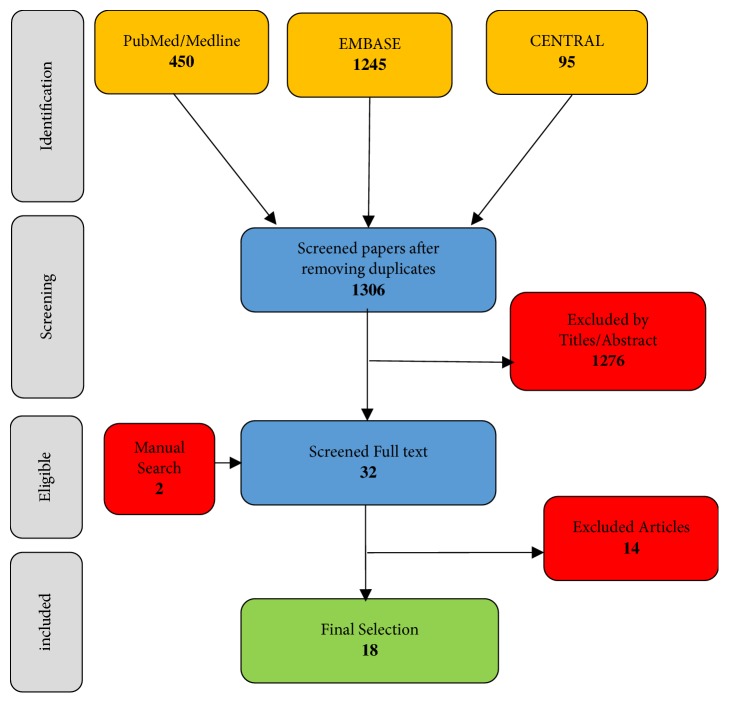
Review process for the titles, abstracts, and full-text reading of the selected references.

The authors of any studies eligible for inclusion in the review, yet without sufficient information, were contacted directly (Figure 1).

### 2.1. Criteria for Inclusion in This Review

#### 2.1.1. Types of Studies

The types of studies included in the research strategy were published or unpublished randomised control trials, case-controlled trials, case series, retrospective studies, and case reports. Papers were obtained from January 2003 to June 2017. Animal studies and those including patients with previous history of radiation therapy to the head and neck regions were excluded. No language restrictions were imposed to the search.

#### 2.1.2. Types of Participants

The review considered studies involving patients who developed MRONJ and subsequently underwent free vascularised osseous tissue transfer and/or osteofasciocutaneous free flap reconstruction. No restriction of age, gender, or ethnic origin was applied. There was no restriction on the minimum number of patients included in the studies.

#### 2.1.3. Types of Interventions

Only free vascularised osseous tissue transfer and/or osteofasciocutaneous free flap reconstruction were considered.

#### 2.1.4. Types of Outcome Measures

*Primary Outcomes*. Primary outcome measures of the review included the success rate of free flap without any restrictions in follow-up. The other considered measures were the frequency of MRONJ recurrence in the free flap or in the surgical residual jaw bone.

*Secondary Outcomes*. The secondary measures of the review entailed perioperative complications and those at follow-up, including the most common cause of the MRONJ and the time during which the patient was treated with the antiresorptive or antiangiogenic drugs prior ONJ.

### 2.2. Data Extracted

Data extracted from the eighteen studies included number of patients, patient sex, and age, predisposing factors for, and localisation of, MRONJ, type of antiangiogenic or antiresorptive drugs and their cumulative dose, clinical indications for the drug or combined therapy, extent of the surgical excision, type of free vascularised tissue reconstruction, free flap failure, immediate complications, follow-up time, and MRONJ recurrence.

All selected papers were carefully read to identify author(s), year of publication, study design, population and treatment characteristics, and number of patients with recurrent MRONJ.

In case of missing information, we contacted the authors and gave them 6 weeks to reply. If the information was still missing we then indicated the missing data as “Not Reported (NR)” in the text and in the tables.

## 3. Results

Results were expressed in descriptive statistics. No randomised controlled clinical trials or case-controlled studies comparing free flap reconstruction after resection in MRONJ patients were found. A total number of 18 articles we included in the study. All the published dates were described in case report (no. 6) and case series (no. 12) from 2008 to 2017 (Table 6). A total of 83 patients, 47 females (56.62%), 19 males (22.89%), and missing information for 20.49% (NR) of the cases, were treated using vascularised osseous tissue transfer and/or osteofasciocutaneous free flap reconstruction.

The most common indications for antiresorptive or antiangiogenic treatment were breast cancer (28.91%), multiple myeloma (22.89%), osteoporosis (14.45%), prostate cancer (9.63%), lung cancer (2.40%), myeloid-leukemia and osteoporosis (1.20%), pain syndrome (1.20%), and NR in the 19.32% of the cases (Table 7). The most common site for MRONJ was the mandible 97.59% and 2.41% in the maxilla (Table 8).

Zoledronate was responsible for the majority of the MRONJ with 42.16 %, then pamidronate 7.22%, alendronate 8.43%, ibandronate 2.40%, and etidronate 1.20%. A combination of the following drugs and the relative incidence percentage were also found responsible:

zoledronate and pamidronate (13.25%);

zoledronate and clodronate (1.20%);

zoledronate and denosumab (1.20%);

pamidronate and denosumab (1.20%);

alendronate, risedronate, and pamidronate (1.20%).

A total of 20.54% patients presented missing information with regard to the type of drug used.

69.90% of the cases missed information on the causes of the MRONJ.

The most commonly utilised vascularised free flap reconstruction was fibula free flap (81.92%), followed by iliac crest (12.04%) and scapula (6.02%).

The most frequent type of resection was subtotal (32.53%), followed by segmental (26.50%) and partial (2.40%). However a large percentage of missing data was found regarding the type of resection (NR 38.57%) (Table 9).

The patients were followed for a period of time ranging from 2 weeks up to 99 months.

Radiographic imaging with CT, cone-beam, and/or orthopantomogram was obtained during follow-up in 95% of the cases.

At follow-up and after free flap reconstruction, recurrence of MRONJ (6.02%) was observed in 5 patients: two of the patients (2.40%) on the contralateral unresected part of the jaw, other two patients (2.40%) on the margin of the resection, and one patient (1.20%) on the grafted flap. The overall free flap failure rate registered was 3.61% (Table 10).

### 3.1. Review Quality Assessment Data

All the studies and data extraction included in the systematic review were qualitative and the risk of bias assessed independently by the authors. The authors used the CARE Checklist for case report and the Modified Delphi Checklist for the case series studies.

In the six case report studies, we identified lack of clarity in many of the thirteen domains, with missing information. We found that the lack of clarity was predominantly on follow-up and diagnostic procedure at the time of follow-up. Hence we concluded the level of bias to be high for all the included case report studies.

In the twelve case series studies, we reported a consistent lack of clarity in some of the seven domains, predominantly regarding the outcome measurement methods. Moreover, we identified some missing information in few other domains; hence we considered the level of bias to be high for all studies

We contacted the authors of these clinical cases to clarify this bias; however we were unable to recover the missing information.

## 4. Discussion

Some antiresorptive drugs such as BP or denosumab have demonstrated to improve the quality of life in patients affected by bone metastasis, osteoporosis, osteopenia, and Paget disease. Additionally, a new antiangiogenic therapy has been successfully used for specific cancer treatments. However, this has remarkably increased the risk of developing MRONJ. This risk is greater in patients who require a higher administration dosage and an intake period greater than 2 years [14, 45, 46].

Moreover, literature has reported that demography, corticosteroid therapy, systemic factors, and genetic factors have been associated with MRONJ. A recent review report showed a wide-ranging MRONJ incidence from 0 to 27.5% in individuals exposed to intravenous BPs, with a mean incidence of 7%, whereas it ranges from 0.1% to 0.06% in oral administrations [47–49].

Etiopathogenesis of MRONJ is not yet fully understood.

Although no gold standard is currently available for the treatment of jaw osteonecrosis, a number of studies debate which MRONJ stage benefits the most from surgical therapy [24, 50]. In general, for early stages of the disease (MRONJ 0 and I) conservative treatments might be sufficient; surgical treatment should be restricted to advanced stages (MRONJ II and III) or after failure of conservative treatments [7, 50].

The majority of researches as well as AAOMS consider conservative treatments as the treatment of choice of MRONJ.

However, there is not a robust evidence from clinical trials as treatment recommendations mostly come from expert opinions and are, therefore, characterised by a low level of evidence [24, 47].

The authors of the 2009 AAOMS position statement recommend reserving resection and immediate reconstruction to patients with stage III of the disease; however, positive outcomes have been noted in patients with stages II and III. Having said that no recommendations were given on which type of reconstruction was to be considered the most predictable [47]. The benefits of surgical management of MRONJ have been extensively debated in literature and radical surgery seems to offer more predictable and curative results. However, surgical treatment of early stages of MRONJ remains controversial [47, 50–52].

Aggressive radical surgery is offered only to symptomatic patients with extensive osteonecrosis, including those who have previously failed conservative treatments [41].

This review has indicated that surgical therapy may represent a treatment option for patients affected by MRONJ stage III resulting in high success rates. Mucke et al. and Caldroney et al. have documented excellent outcomes in treating patient affected by MRONJ stage III in large cohort studies [30, 43]. Since 2008 microvascular reconstruction of the jaw has been documented as a viable option for MRONJ. This systematic review confirmed that microsurgical reconstruction therapy represents a feasible alternative in case of treatment escalation.

Even though the majority of papers included in this study were case reports and small case studies, the outcome of free flap treatment has been promising with a significant low recurrence of MRONJ and minimal surgical complications [25, 26, 28–43].

The MRONJ recurrence rate found by this systematic review was 6.02% (5 patients). The predominant recurrence sites were the contralateral unresected part of the jaw (2 cases) and the margin of the resection (2 cases), both bearing an overall recurrence rate of 2.40%. Just one case of recurrence was found on the vascular reconstruction.

Infection was the most frequent complication found with 6.02% incidence. The overall free flap success rate was 96.39%. Three free flaps failed during a follow-up period ranging from 2 weeks up to 99 months.

Amongst all the types of reconstruction, free flap fibula was the most chosen, followed by iliac crest and scapula with success rates, respectively, of 97.60%, 98.80%, and 100%.

Antiresorptive drugs were explicitly discontinued in only three studies out of the eighteen, while no mention was reported in the remaining studies [31, 33, 41]. It is unclear if the discontinuation strategy leads to a better surgical outcome due to the long skeletal life of some antiresorptive drugs.

In line with the growing body of literature, our findings confirm positive results in treating patients with MRONJ using free flaps microvascular reconstruction. In order to obtain a possible resolution of MRONJ, patients with reasonable life expectancy should be considered for microvascular flap reconstruction after aggressive resection of the diseased bone.

## 5. Conclusion

MRONJ is a significant adverse effect amongst patients under antiresorptive agents. Although MRONJ pathogenesis remains unclear, significant progress has been made with respect to the diagnosis and staging of the disease, as well as with risk-reduction strategies and treatments. This systematic review based on multiple-reviewer quality assessment criteria was only able to select articles that meet Level 4 of the Oxford Evidence-based medicine scale. Due to the nature of the MRONJ incidence and the critical condition of the patients affected by the primary disease, it is difficult to improve the quality of evidence unless a common effort is applied. Therefore, the authors believe that additional quality studies, such as control multicentre studies or case-controlled studies, are necessary to support the hypothesis of this study.

## Figures and Tables

**Table 1:** Antiresorptive drugs used in oncologic and nononcologic patients. Btl: bottle; IM: intramuscular; IV: intravenous; MM: multiple myeloma; PO: orally; SC: subcutaneous; SRE: skeletal-related event; Tab: tablet.

**Pharmacologic active ingredient **	**Formulation**	**Route of administration**	**Indication and frequency**
Alendronic acid (sodium salt)	Tab 70 mg Tab 10 mg	PO	Treatment of postmenopausal osteoporosis (70 mg/week) Treatment of osteoporosis in men (70 mg/week) Treatment and prevention of osteoporosis induced by glucocorticoids (70 mg/week)

Alendronic acid + cholecalciferol	Tab 70 mg/5600 UI	PO	Treatment of postmenopausal osteoporosis in patients with unsupplemented vitamin D deficit (70 mg/week)

Ibandronic acid (monosodium salt monohydrate)	Tab 50 mg Btl 6 mg/6 ml Tab 150 mg Btl 3 mg/3 ml	PO IV PO IV	Prevention of SREs in breast cancer patients with bone metastases (50 mg/day p.o. or 6 mg every 3–4 weeks iv.) Treatment of hypercalcemia of malignancy Treatment of postmenopausal osteoporosis in patients at high risk of fracture (150 mg/4 weeks p.o. or 3 mg every 3 months iv.)

Neridronate acid (sodium salt)	Btl 25 mg/2 ml Btl 100 mg/8 ml	IV/IM. IV	Osteogenesis imperfecta (2 mg/kg/3 months) Paget's bone disease (different schedules)

Pamidronic acid (disodium salt)	Btl 15 mg/5 ml Btl 30 mg/10 ml Btl 60 mg/10 ml Btl 90 mg/10 ml	IV	Prevention of SREs in breast cancer patients with bone metastases or MM with bone lesions (60–90 mg every 3–4 weeks) Treatment of hypercalcemia of malignancy

Zoledronic acid (monohydrate)	Btl 4 mg/5 ml Btl 5 mg/100 ml	IV IV	Prevention of SREs in cancer patients with bone metastases or MM (4 mg every 3–4 weeks). Treatment of hypercalcemia of malignancy Treatment of osteoporosis in postmenopausal women, in men at increased risk of fracture, including those with a recent hip fracture from minor trauma (5 mg once per year) Treatment of bone Paget's disease

Denosumab	Btl 120 mg Btl 60 mg	SC SC	Prevention of SREs in cancer patients with bone metastases (120 mg every 4 weeks) Treatment of hypercalcemia of malignancy. Osteoporosis (60 mg sc. every 6 months)

**Table 2:** Main antiangiogenic drugs used (IV: intravenous; MM: multiple myeloma; PO: orally; SC: subcutaneous; Btl: bottle; Tab: tablet).

**Pharmacologic active ingredient **	**Formulation**	**Route of administration**	**Indication and frequency**
Bevacizumab	Btl 400 mg Btl 100 mg	IV	Metastatic breast cancer (10 mg/kg every 2 weeks or 15 mg/kg every 3 weeks); colorectal cancer (5 mg/kg or 10 mg/kg every 2 weeks); lung/ovarian cancer (7.5 mg/kg or 15 mg/kg every 3 weeks); renal cell cancer (10 mg/kg every 2 weeks); glioblastoma (10 mg/kg every 2 weeks)

Sunitinib	Tab 12.5 mg	PO	Renal cell cancer, GISTs and neuroendocrine tumors (50 mg/day for 4 weeks)

Sorafenib	Tab 200 mg	PO	Renal cell cancer (800 mg/day)

Pazopanib	Tab 200 mg Tab 400 mg	PO	Renal cell cancer (200–800 mg/day)

Thalidomide	Tab 50 mg	PO	Myeloma (400 mg/day for 6 weeks)

Lenalidomide	Tab 5, 10, 15 and 25 mg	PO	Myeloma (tailored doses)

Everolimus	Tab 5 and 10 mg	PO	Renal cell cancer, breast cancer (10 mg every day)

Temsirolimus	Btl 30 mg	IV	Renal cell cancer (25 mg every week)

**Table 3:** Drug-related risk factor of osteonecrosis of the jaw in the cancer population according to Campisi et al. 2011 [11].

**Risk Factor **	**Strenght**
Zoledronate vs Other Bisphosphonate	+++

Intravenouse vs Oral Bisphosponate	++

Bisphosphonate cumulative dose	+++

Bisphosphonate duration of treatment	+++

Anti-angiogenic drugs	++

Denosumab	++

Chemotherapy	-/+

Thalilomide	+/-

**Table 4:** MRONJ staging according the AAOMS [7].

**Stage**	**MRONJ clinical findings **
At risk category	No apparent necrotic bone in patients who have been treated with either oral or IV bisphosphonates

Stage 0	No clinical evidence of necrotic bone, but non-specific clinical findings, radiographic changes and symptoms

Stage I	Exposed and necrotic bone, or fistulae that probes to bone, in patients who are asymptomatic and have no evidence of infection

Stage II	Exposed and necrotic bone, or fistulae that probes to bone, associated with infection as evidenced by pain and erythema in the region of the exposed bone with or without purulent drainage

Stage III	Exposed and necrotic bone or a fistula that probes to bone in patients with pain, infection, and one or more of the following: exposed and necrotic bone extending beyond the region of alveolar bone, (i.e., inferior border and ramus in the mandible, maxillary sinus and zygoma in the maxilla) resulting in pathologic fracture, extra-oral fistula, oral antral/oral nasal communication, or osteolysis extending to the inferior border of the mandible of sinus floor

**Table 5:** Systematic review currently published and their limitations.

**Systematic Review **	**Limitation**
Sacco et al. (2011) [27]	English literature limited search; Single Electronic database search.

Vercruysse et al. (2014) [25]	Search Limited to BRONJ and or bisphosphonate related necrosis; No mentioning to language limitation; Review based on a single reviewer selection of articles

Neto et al. (2016) [26]	Single Electronic database search; Search Limited to BRONJ and or bisphosphonate related necrosis; No mentioning to language limitation; No mentioning reviewer involved in the search strategy.

**Table 6:** Study selected with total number of patient treated.

**Study**	**Type of study**	**Patients number**
Engroff and Kim (2007) [28]	Case series	2

Ferrari et al. (2008) [29]	Case Report	1

Mücke et al. (2009) [30]	Case series	2

Nocini et al. (2009) [31]	Case series	7

Seth et al. (2010) [32]	Case series	11

Bedogni et al. (2011) [33]	Case series	3

Pautke et al. (2011) [34]	Case report	1

Bittner et al. (2012) [35]	Case report	1

Ghazali et al. (2013) [36]	Case report	1

Hanasono et al. (2013) [37]	Case series	11

Horta et al. (2014) [38]	Case series	1

Spinelli et al. (2014) [39]	Case series	8

Vercruysse et al. (2014) [25]	Case series	3

Kim et al. (2015) [40]	Case series	4

Mücke et al. (2016) [41]	Case series	14

Neto et al. (2016) [26]	Case report	1

Sotsuka et al. (2016) [42]	Case report	1

Caldroney et al. (2017) [43]	Case Series	11

**Table 7:** Preoperative pharmacological analysis: type of drugs, indication for drug therapy, and time of drug exposure. ZOL: zoledronate; ALD: alendronate; PMT: pamidronate; COL: clodronate; DZM: denosumab; IBA: ibandronate; ETI: etidronate; mth: months; RSD: risedronate; NR: not reported.

**Study**	**Type of drug**	**Indication for drug therapy**	**time of drug exposure**
Engroff and Kim (2007) [28]	PMT (x 1 case) ZOL (x 1 case)	Brest Cancer (x 2 cases)	NR

Ferrari et al. (2008) [29]	PMT + ZOL	Multiple Myeloma	21 mth (PMT) 3 mth (ZOL) discontinue therapy

Mücke et al. (2009) [30]	ZOL (x 2 cases)	Brest cancer (x 1 case); Multiple Myeloma (x 1 case)	50mth (ZOL) 36 mth (ZOL)

Nocini et al. (2009) [31]	PMT and ZOL (x 5 cases) ZOL (x 2 cases)	Brest Cancer (x 5 cases); Prostate Cancer (x 1 case); Myeloid leukaemia and Osteoporosis (x 1 case)	NR

Seth et al. (2010) [32]	ZOL (x 6 cases) ALD (x 2 cases) IBA (x 2 cases) ETI (x 1 case)	Brest Cancer (x 5 cases); Prostate Cancer (x 2 cases); Multiple Myeloma (x 2 cases); Osteoporosis (x 2 cases)	NR

Bedogni et al. (2011) [33]	NR	NR	NR

Pautke et al. (2011) [34]	ZOL	Prostate Cancer	40 mth

Bittner et al. (2012) [35]	ZOL and PMT	Pain syndrome	12 mth (ZOL) 3 mth (PMT)

Ghazali et al. (2013) [36]	ALD	Osteoporosis	84 mth (ALD)

Hanasono et al. (2013) [37]	ZOL (x 9 cases) PMT (x 2 cases)	Multiple Myeloma (x 5 cases); Breast Cancer (x 2 cases); Prostate Cancer (x 2 cases); Osteoporosis (x 2 cases)	NR

Horta et al. (2014) [38]	ZOL	Lung Cancer	36 mth (ZOL)

Spinelli et al. (2014) [39]	ZOL (x 3 cases) PMT (x 3 cases) ZOL and PMT (x 2 cases)	Multiple Myeloma (x 4 cases); Brest Cancer (x 3 cases); Prostate Cancer (x 1 case)	1 x 27 mth (ZOL) 1 x 21 mth (ZOL) 1 x 35 mth (ZOL 1 x 22 mth (PMT) 1 x 30 mth (PMT) 1 x 19 mth (PMT 1 x 25 mth (ZOL and PMT) 1 x 17 mth (ZOL and PMT)

Vercruysse et al. (2014) [25]	1 x ZOL 1 x ZOL + PMT 1 x ZOL + CLO	Multiple Myeloma (x 2 case); Brest Cancer (x 1 case)	1 x 22mth (ZOL) 1x 12 mth (PMT) + 26 mth (ZOL) 1x 96 mth (CLO) + 29mth (ZOL)

Kim et al. (2015) [40]	ALD (x 2) ALD + RSD + PMT (x 1) ZOL + PMT (x 1)	Osteoporosis (x 3) Multiple Myeloma (x 1)	1 x 48 mth (ALD) 1 x 120 mth (ALD 1 x 24 mth (ALD + RSD +PMT) 1 x 30mth (ZOL +PMT)

Mücke et al. (2016) [41]	NR	NR	NR

Neto et al. (2016) [26]	ZOL	Lung Cancer	36 mth (ZOL)

Sotsuka et al (2016) [42]	ZOL	Brest Cancer	59 mth (ZOL)

Caldroney et al. (2017) [43]	7 x ZOL 2 x ALD 1 x ZOL + DZM 1 x PMT + DZM	Brest Cancer (x 4 cases) Osteoporosi (x 3 cases) Multiple Myeloma (x 3 cases) Prostate Cancer (x 1 case)	NR

**Table 8:** Preoperative epidemiologic analysis (age, sex, predisposing factors, and site of the necrosis involved). M: male; F: female; NR: not reported.

**Study**	**Type of study**	**Patients number**	**Age/Sex**	**Trigging cause**	**Site of the necrosis involved**
Engroff and Kim (2007) [28]	Case series	2	64 (F); 49 (F)	Dental extraction (x 2 cases)	Mandible (x 2 cases)

Ferrari et al. (2008) [29]	Case report	1	66 (M)	NR	Mandible

Mücke et al. (2009) [30]	Case series	2	48 (F); 60 (F)	Dental Extraction (x 1 case); Spontaneous (x 1 case)	Mandible (x 2 cases)

Nocini et al. (2009) [31]	Case series	7	NR (six F); (one M)	Oral surgery (x 5 cases); Infection (x 2 cases)	Mandible (x 7 cases)

Seth et al. (2010) [32]	Case series	11	68 (M); 56 (F); 50 (F); 72 (F); 48 (F); 71 (F); 67 (F); 60 (F); 51 (F); 72 (M); 60 (F)	NR	Mandible (x 11 cases)

Bedogni et al. (2011) [33]	Case series	3	NR	NR	Mandible (x 2 cases); Maxilla (x 1 case)

Pautke et al. (2011) [34]	Case report	1	76 (M)	Dental extraction	Mandible

Bittner et al. (2012) [35]	Case report	1	41 (F)	Dental extraction	Mandible

Ghazali et al. (2013) [36]	Case report	1	82 (F)	Dental extraction	Mandible

Hanasono et al. (2013) [37]	Case series	11	63 (F); 57 (M); 65 (M); 75 (F); 72 (M); 68 (M); 60 (F); 64 (F); 70 (F); 75 (F); 67 (F)	NR	Mandible (x 11 cases)

Horta et al. (2014) [38]	Case series	1	54 M	Spontaneous	Mandible

Spinelli et al. (2014) [39]	Case series	8	73 (M); 77 (F); 64 (F); 53 (F); 62 (M); 68 (F); 57 (M); 64 (F)	Dental extraction (x 3 cases); Spontaneous x 5	Mandible (x 8 cases)

Vercruysse et al. (2014) [25]	Case series	3	54 (F); 70 (F); 64 (F)	Dental extraction (x 1 case); Spontaneous (x 2 cases)	Mandible (x 3 cases)

Kim et al. (2015) [40]	Case series	4	69 (F), 68 (F), 62 (F), 70 (M)	NR	Mandible (x 4)

Mücke et al. (2016) [41]	Case series	14	NR	NR	Mandible (x 14 cases)

Neto et al. (2016) [26]	Case report	1	58 (M)	Spontaneous	Mandible

Sotsuka et al. (2016) [42]	Case report	1	50 (F)	NR	Maxilla

Caldroney et al. (2017) [43]	Case series	11	56 (F); 65 (F); 60 (F); 61 (F); 65 (F); 64 (F); 68 (M); 67 (M); 73 (M); 72 (F); 73 (M).	NR	Mandible (x 11 cases)

**Table 9:** Operative analysis: type of surgery, type of free flap, flap failure, immediate postoperative complications (FFF: fibula Free flap; ICFF: iliac crest free flap; SFF: scapula free flap).

**Study**	**Type of surgery**	**Type of free flap**	**Flap failure**	**Immediate post-operative complications**
Engroff and Kim (2007) [28]	2 x Segmental	2 x FFF	0	Small Neck hematoma in one patient

Ferrari et al. (2008) [29]	Sub-total	1x FFF	0	0

Mücke et al. (2009) [30]	2 x Segmental	1 x FFF; 1x ICFF	0	0

Nocini et al. (2009) [31]	7 x Subtotal	7 x FFF	0	Rupture of mini-plate in one patient

Seth et al. (2010) [32]	NR	11 x FFF	0	Prolonged infection in one patient; Fistula and infection in three patients.

Bedogni et al. (2011) [33]	NR	3 x FFF	1 (a year later)	0

Pautke et al. (2011) [34]	Segmental	1 x ICFF	0	Fistula resolved with removal of plate

Bittner et al. (2012) [35]	Segmental	1 x SFF	0	0

Ghazali et al. (2013) [36]	Segmental	1 x FFF	0	Sinus bradycardia

Hanasono et al. (2013) [37]	6 x subtotal 5 x segmental	11 x FFF	1	Hematoma in one patient; Pneumonia in one patient; Deep vein thrombosis in one patient; Small bowel obstruction in one patient. All complications occurred in FFF

Horta et al. (2014) [38]	1 x segmental	1 x FFF	0	0

Spinelli et al. (2014) [39]	8 x subtotal	8 x FFF	0	0

Vercruysse et al. (2014) [25]	2 x Partial; 1 x Segmental	3 x ICFF	1 (segmental- 16 days later)	1 (failure)

Kim et al. (2015) [40]	NR	4 x FFF	0	0

Mücke et al. (2016) [41]	NR	9 x FFF 5 x ICFF	NR	NR

Neto et al. (2016) [26]	1 x segmental	1 x FFF	0	0

Sotsuka et al. (2016) [42]	NR	1 x FFF	0	0

Caldroney et al. (2017) [43]	6 x segmental 5 x sub total	4 x SFF 7 x FFF	0	Two cases with wound infection and dehiscence and one case the plate was removed. (3 different patients). One FFF and two SFF

**Table 10:** Complications during follow-up time.

**Study**	**Follow-up time**	**Complications during follow-up (included plate removal)**	**MRONJ recurrence**	**Site of recurrence**
Engroff and Kim (2007) [28]	2x12 months	0	Recurrence in one patient	Contralateral

Ferrari et al. (2008) [29]	1 x 12 months	Plate removal	0	0

Mücke et al. (2009) [30]	2x 12 months	0	0	0

Nocini et al. (2009) [31]	1x 6 months 1 x 16 months 1 x 23 months 1 x 24 months 1 x 19 months 1 x 33 months 1 x 34 months	0	Recurrence in one patient	Margin of the resection

Seth et al. (2010) [32]	1 x 10.0 months 1x 0.5 months 1x 30.8 months 1x 21.4 months 1x 17.8 months 1x 23.7 months 1x 10.6 months 1 x 14.2 months 1x 13.9 months 1x 12.2 months 1x 6.1 months	0	0	0

Bedogni et al. (2011) [33]	NR	failure of the FFF 1 year later	0	0

Pautke et al. (2011) [34]	NR	plate removal	Recurrence in one patient	On the free flap

Bittner et al. (2012) [35]	NR	0	0	0

Ghazali et al. (2013) [36]	24 months	0	0	0

Hanasono et al. (2013) [37]	1 x 13.3 months 1x 20.1 months 1x 77.0 months 1x 23.8 months 1x 11.4 months 1 x 9.1 months 1x 9.1 months 1x 9.1 months 1x 8.1 months 2 x 3.0 months	0	0	0

Horta et al. (2014) [38]	1 x 12 months	0	0	0

Spinelli et al. (2014) [39]	1x 21.7 months 1x 25.1 months 1x 28.4 months 1x 32.2 months 1x 37 months 1x 28.4 months 1x 25.1 months 1x 32.9 months	0	0	0

Vercruysse et al. (2014) [25]	1x 36 months 1x 65 months 1x 76 months	Plate removal in one patient	Recurrence in one patient	Contralateral

Kim et al. (2015) [40]	1 x 99 months 1 x 18 months 1 x 12 months 1 x 7 months	Fracture of plate in one patient	0	0

Mücke et al. (2016) [41]	34.25 ± 33.3 months	-	Recurrence in one of the patient	Margin of the flap

Neto et al. (2016) [26]	1 x 48 months	0	0	0

Sotsuka et al. (2016) [42]	NR	0	0	0

Caldroney et al. (2017) [43]	3 x 6 months 2 x 44 months 1 x 69 months 1 x 36 months 1 x 28 months 1 x 10 months 1 x 17 months 1 x 11 months	Plate removal in one patient	0	0
